# The Influence of Personality Traits on Reported Adherence to Medication in Individuals with Chronic Disease: An Epidemiological Study in West Sweden

**DOI:** 10.1371/journal.pone.0018241

**Published:** 2011-03-28

**Authors:** Malin Axelsson, Eva Brink, Jesper Lundgren, Jan Lötvall

**Affiliations:** 1 Krefting Research Centre, Internal Medicine, Institute of Medicine, Sahlgrenska Academy, University of Gothenburg, Gothenburg, Sweden; 2 Department of Nursing, Health and Culture, University West, Trollhättan, Sweden; 3 Institute of Health and Care Sciences, Sahlgrenska Academy, University of Gothenburg, Gothenburg, Sweden; 4 Department of Psychology, University of Gothenburg, Gothenburg, Sweden; L' Istituto di Biomedicina ed Immunologia Molecolare, Consiglio Nazionale delle Ricerche, Italy

## Abstract

**Background:**

Limited research exists exploring the influence of personality on adherence behaviour. Since non-adherence is a major obstacle in treating prevalent chronic diseases the aim was to determine whether personality traits are related to reported adherence to medication in individuals with chronic disease.

**Methodology/Principal Findings:**

Individuals with chronic disease (n = 749) were identified in a random population sample of 5000 inhabitants aged 30–70 in two municipalities in West Sweden. Data on five personality traits, Neuroticism, Extraversion, Openness to experiences, Agreeableness, and Conscientiousness, and medication adherence behaviour was collected by questionnaires. Statistical analyses resulted in a negative relationship between Neuroticism and medication adherence (P<0.001), while both Agreeableness (P<0.001) and Conscientiousness (P<0.001) were positively related to adherence. At high levels of Conscientiousness, low adherence was related to higher scores in Neuroticism. At high levels of Agreeableness, low adherence was related to low scores in Conscientiousness and high scores in Openness to experiences.

**Conclusions:**

This study demonstrated that multiple personality traits are of significant importance for adherence behaviour in individuals with chronic disease. The findings suggest that several personality traits may interact in influencing adherence behaviour. Personality traits could putatively be used to focus efforts to educate and support patients with high risk of low medical adherence.

## Introduction

The World Health Organization (WHO) has highlighted the issue of non-adherence to long-term treatment, which is a major obstacle in treating prevalent chronic diseases, such as asthma, hypertension, and diabetes [1]. Insufficient adherence to treatment can lead to unmet treatment expectations resulting in inadequate disease control [2]–[5]. It is recognised that adherence behaviour is closely related to the efficacy of a prescribed treatment [6]. Decades of research into treatment adherence have defined many determinants of adherence behaviour [1]. However, few studies have explored the associations between adherence behaviour and personality. One model that is commonly used to describe personality is the five-factor model (FFM) of personality, also known as Big Five, which describes personality according to traits: Neuroticism, Extraversion, Openness to experiences, Agreeableness, and Conscientiousness. The FFM thus represents a structure of personality traits consisting of five broad bipolar domains or dimensions that are defined by clusters of interrelated specific traits. Neuroticism measures degrees of emotional stability, Extraversion describes degrees of interpersonal interactions, Openness to experience estimates willingness to accept novel ideas, Agreeableness estimates the quality of interpersonal orientation, and Conscientiousness estimates motivation in goal-directed behaviour [7]. The theoretical background of the FFM stipulates that our personality is biologically influenced, which could explain its relative stability over time after the age of 30. Personality should be regarded as a predisposition and not as a fixed prediction of certain behaviour, because we usually adapt our actions depending on the existing social or environmental situation or emotional state. Thus, although personality could be useful in predicting behaviour across time, such behaviour can be influenced [8]. The strength with the FFM lays in its descriptions of personality [9] and in its comprehensiveness as personality taxonomy when being tested in relation to other personality inventories [10]. The limitation could be its sparse explanations of how personality is associated with health and that the FFM does not include situational factors. Nevertheless, it is argued that the FFM could serve as an incentive for research on personality and health that could yield a coherent ground for the present research area [9].

Previous studies, using clinically recruited patient cohorts, have suggested that adherence behaviour can be related to personality traits. Perhaps the best example of an association between a personality trait and adherence behaviour is Conscientiousness, which has been positively related to medication adherence in individuals undergoing renal dialysis [11], treatment for AIDS [12], and treatment with cholesterol-lowering drugs [13]. Other studies have shown varying results [14]–[18]; one study focusing on women living with HIV/AIDS failed to show any clear associations between FFM personality traits and medication adherence [19]. To date, we are aware of only one epidemiological study that has investigated associations between personality traits and medication adherence in chronic disease in a random population sample; this study focused only on young adults, aged approximately 22 years [20]. Chronic disease is more prevalent in an older population and personality traits have been reported to be more stable after 30 years of age. Also, the effect of personality traits on adherence behaviour is not fully explored, therefore the aim of the current study was to determine whether a relationship exists between FFM personality traits and reported adherence to medication in a random population of adults aged 30–70.

## Materials and Methods

### Participants

**Figure 1:**
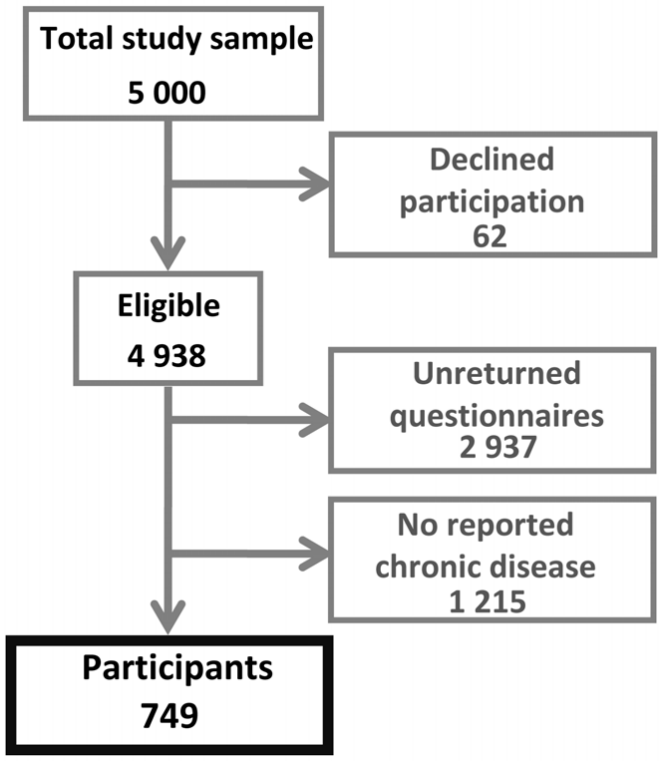
Sampling procedure.

Postal questionnaires, including pre-paid reply envelopes, were sent to 5000 randomly selected individuals between the ages of 30–70 years in West Sweden during the spring of 2009. The population had an equal gender ratio and contact information was provided by the Swedish Population Register. Administration of the questionnaires was performed by an external company. In addition to the original questionnaire, two reminders were sent to non-responders. The response rates to the questionnaire and the two reminders were 19.4%, 11.5% and 9.1%, respectively, which gave a total study group of 2001 individuals. 749 individuals who reported diagnosed chronic disease were selected for the present study. The sampling procedure is presented in Figure 1.

A non-response analysis, consisting of a structured telephone interview, was carried out by one interviewer during two weeks in April 2010 on 120 randomly selected individuals who did not return questionnaires after the second reminder. 36 individuals were unreachable after five attempts during both day and evening hours and 28 declined participation. 56 individuals gave verbal consent to take part in the analysis. They were verbally informed that their answers would be registered in a confidential database only to be used for the purpose of research.

### Measurements

#### Main study

The questionnaires contained socio-demographic questions including questions on prevalence of chronic disease and prescribed medication. The Medication Adherence Report Scale (MARS) was used to measure adherence on a continuous scale. The MARS contains five items (“*I forget to take them*”, “*I alter the dose*”, “*I stop taking them for a while*”, “*I decide to miss out a dose*”, and “*I take less than instructed*”) with a five-point response scale ranging from “always” to “never” with a total score of 25. The higher scores, the better adherence [21]. Cronbach's alpha for MARS was 0.805 in the present study.

The Neuroticism, Extraversion, and Openness to experience Five Factor Inventory (NEO-FFI) was used to assess the five personality traits on a continuous scale. The NEO-FFI contains 60 items (12 for each personality trait) with five responses ranging from “strongly disagree” to “strongly agree” [7]. Cronbach's alpha values for the NEO-FFI in the present study were: Neuroticism, 0.875; Extraversion, 0.823; Openness to experiences, 0.685; Agreeableness, 0.715; Conscientiousness, 0.790.

#### Non-response study

The structured telephone interview contained eight key questions. The following three questions were derived from the original questionnaires: *“Do you have a chronic/long-term disease that is diagnosed by a clinician?”*, *“What is your age?”, “What is your educational level?”* The remaining five questions were derived from the NEO-FFI and they were used to test the hypothesis that personality scores differ between responders and non-responders. One item from each of the five personality traits was selected from the NEO-FFI. The selection of items was preceded by inter-reliability tests for each personality scale to identify the item of most significance for each scale. Analyses were performed using the Statistical Package for the Social Sciences (SPSS) version 17 for Windows.

### Statistical analysis

#### Main study

Descriptive statistics, including, frequencies, percentages, means and standard deviations were calculated for all scores. Differences in socio-demographic variables and adherence scores between men and women were analysed with Fisher's permutation test. Univariate associations between personality traits and adherence behaviour were tested by Pitman's permutation test [22]. Personality variables with a P-value <0.05 were entered in a multiple linear regression model. Based on assumptions of non-linear relationships between personality and adherence, multiple regression analyses with spline function were conducted with MARS as dependent variable and personality traits as independent predictors. The spline function is a piecewise function with linear pieces at the ends and quadratic splines in the intermediate intervals. In contrast to a linear regression, the quadratic regression makes the line more “flexible” and its shape is depending on the data points. This due to the so called knots, i.e. the juncture between the intervals, which were set at the 10-, 50- 90- percentile points. The number of knots was adjusted to data [23].

#### Non-response study

Frequencies, percentages, means and standard deviations were calculated. In order to compare the non-responders with the original sample (n = 2001), the Chi-square test was used to compare nominal data i.e. presence of chronic illness yes/no, Mann-Whitney test was used to compare the ordinal variable “education level”, and Student's *t*-test was used for the continuous variables, age and personality traits [24].

### Ethical approval

Ethical approval was granted by the regional research ethics board at the University of Gothenburg.

## Results

The study group contained more women than men (57% vs. 43%). The men were older, more frequently self-employed, and had higher income but lower level of education than women (Table 1). There was no significant difference in MARS scores between men and women. Women scored higher on all personality traits compared with men (Table 2).

**Table 1:** Socio-demographic description of the sample (n = 749).

Variable	All n (%)	Men n (%)	Women n (%)	P
**Sex**	749	322 (43%)	427 (57%)	0.001
**Mean age in years (SD)**	53.59 (11.09)	54.60 (11.21)	52.83 (10.96)	0.034
**Education level ≤9 years**	205 (27.4%)	98 (30.4%)	107 (25.1%)	0.0086
**Livelihood**				
Employee	438 (58.5%)	181 (56.2%)	257 (60.2%)	0.28
Self-employed	34 (4.5%)	24 (7.5%)	10 (2.3%)	0.0017
Pensioner	196 (26.2%)	90 (28%)	106 (24.8%)	0.30
Other	77 (10.2%)	26 (8%)	51 (12%)	-
**Monthly own income**				0.001
<15.000 SEK	186 (24.8%)	49 (15.2%)	137 (32.1%)	
15.000–25.000 SEK	286 (38.2%)	106 (32.9%)	180 (42.2%)	
25.000–40.000 SEK	195 (26%)	120 (37.3%)	75 (17.6%)	
>40.000 SEK	34 (4.5%)	28 (8.7%)	6 (1.4%)	
**Chronic diseases**				
Asthma	86 (11.5%)	28 (8.7%)	58 (13.6%)	0.048
COPD *	12 (1.6%)	3 (0.9%)	9 (2.1%)	0.26
Heart failure	3 (0.4%)	1 (0.3%)	2 (0.5%)	0.30
Hypertension	232 (31%)	101 (31.4%)	131 (30.7%)	0.30
Angina pectoris	26 (3.5%)	13 (4%)	13 (3%)	0.30
Cardiac infarction	29 (3.9%)	18 (5.6%)	11 (2.6%)	0.039
Stroke	20 (2.7%)	8 (2.5%)	12 (2.8%)	0.30
Rheumatoid arthritis	31 (4.1%)	6 (1.9%)	25 (5.9%)	0.0055
Diabetes	74 (9.9%)	43 (13.4%)	31 (7.3%)	0.0066
Depression	102 (13.6%)	34 (10.6%)	68 (15.9%)	0.036
Allergic rhinitis	123 (16.4%)	59 (18.3%)	64 (15%)	0.24
Other	327 (43.7%)	123 (38.2%)	204 (47.8%)	0.0097
**Regular treatment**				
Oral	486 (64.9%)	201 (62.4%)	285 (66.7%)	0.15
Inhalation	76 (10.1%)	24 (7.5%)	52 (12.2%)	0.044
Injection	36 (4.8%)	25 (7.8%)	11 (2.6%)	0.0019
Other	100 (13.4%)	40 (12.4%)	60 (14.1%)	0.30
**Irregular treatment**				
Oral	249 (33.2%)	98 (30.4%)	151 (35.4%)	0.18
Inhalation	103 (13.8%)	37 (11.5%)	66 (15.5%)	0.14
Injection	14 (1.9%)	10 (3.1%)	4 (0.9%)	0.058
Other	57 (7.6%)	19 (5.9%)	38 (8.9%)	0.16

*Chronic Obstructive Pulmonary Disease.

**Table 2:** Mean scores and standard deviations (SD) of personality traits and MARS¤.

Variable	Total sample (n = 749) mean (SD)*	Men (n = 322) mean (SD)*	Women (n = 427) mean (SD)*	P
**MARS** ¤	22.73 (2.94)	22.52 (3.00)	22.89 (2.88)	0.085
**Neuroticism**	17.12 (8.67)	15.93 (8.32)	18.02 (8.82)	0.0011
**Extraversion**	28.86 (6.89)	27.98 (6.76)	29.53 (6.91)	0.0022
**Openness to Experiences**	26.21 (6.03)	24.34 (5.71)	27.62 (5.88)	0.001
**Agreeableness**	34.36 (5.28)	32.85 (5.37)	35.49 (4.92)	0.0001
**Conscientiousness**	33.88 (5.86)	33.36 (5.68)	34.27 (5.97)	0.036

¤ **= **Medication Adherence Report Scale,

* = Standard deviation.

Associations between the five personality traits and MARS are presented in Table 3. Neuroticism was found to negatively influence MARS, which indicates that individuals who rated high on this personality trait were more disposed to a non-adherent behaviour. Both Agreeableness and Conscientiousness were positively associated with MARS, indicating that individuals scoring high on these personality traits tended to be more adherent to treatment. There were no linear associations between the personality traits Openness to experiences and Extraversion and MARS.

**Table 3:** Univariate correlations between personality traits, age and MARS (n = 749).

Variable	MARS¤	P
**Neuroticism**	−0.155	0.001
**Extraversion**	0.012	0.30
**Openness to experiences**	−0.064	0.082
**Agreeableness**	0.129	0.001
**Conscientiousness**	0.162	0.001
**Age**	0.238	0.001

¤ = Medication Adherence Report Scale.

**Figure 2:**
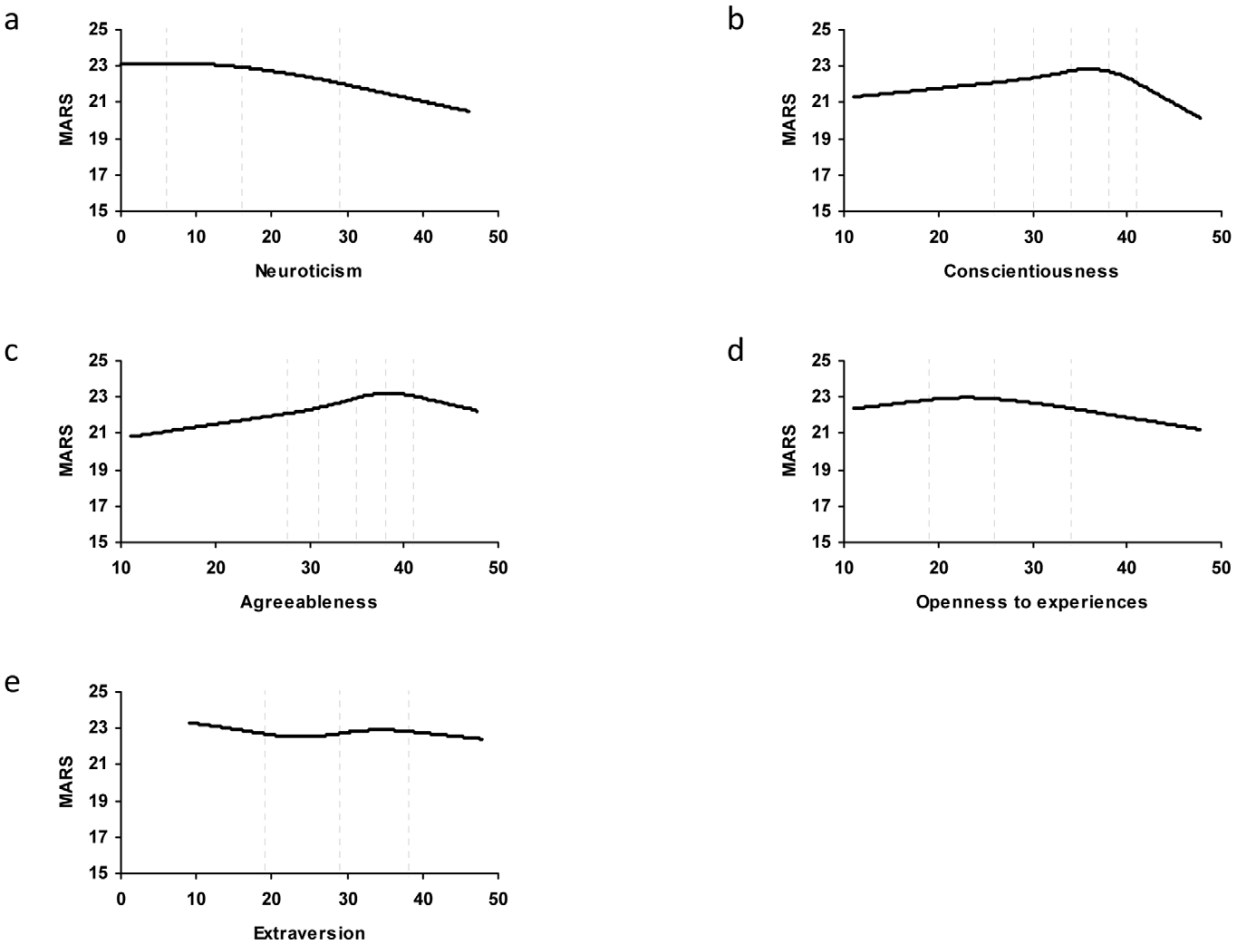
Multiple regression with a spline function with MARS as dependent variable. The vertical dashed lines show the percentile points. Neuroticism: 6, 16, 29. Conscientiousness: 26, 30, 34, 38, 41. Agreeableness: 27.6, 31, 35, 38, 41. Openness to experiences: 19, 26, 34.

A multiple regression analysis (*F*  = 10.393, P<0.001) with MARS as an independent variable and the three personality traits that had a significant relationship to MARS in the univariate analyses (Table 3), showed that only 4% (adjusted *R* ^2^ = 0.036) of the variance in MARS could be directly explained by each personality trait (Table 4). This result led to the assumption that non-linear relationships between personality traits and MARS can exist. A multiple regression analysis with a spline function indicated a negative relationship between Neuroticism and MARS across the entire scale (Figure 2a). However, the relationship between Conscientiousness and MARS was non-linear, with an inflection point at 37 on the Conscientiousness scale, after which MARS scores decreased substantially (Figure 2b). The relationship between Agreeableness and MARS was also non-linear with an inflection point at 40, after which MARS scores clearly decreased (Figure 2c). This association signified that individuals who rated very high on Conscientiousness or Agreeableness were associated with both higher and lower MARS scores.

**Table 4:** Multiple regression model with MARS¤ as dependent variable (n = 749).

Variables	β	Standard error	P
**Constant**	20.043		0.001
**Neuroticism**	−.029	1.050	0.035
**Agreeableness**	.043	.014	0.044
**Conscientiousness**	.051	.021	0.013

**¤** Medication Adherence Report Scale.

A detailed exploration of the group that scored high for Conscientiousness revealed a mixed population with both high and low scores on MARS. Within this group, individuals that rated high on Conscientiousness (≥37) but low on MARS (≤19) scored significantly higher on Neuroticism than those scoring high on MARS (Table 5). Similarly, a detailed investigation of individuals that rated 40 or higher on Agreeableness revealed a mixed population with both high and low scores on MARS, and the individuals scoring high on MARS scored low on Openness to experiences and high on Conscientiousness compared to those scoring low on MARS (Table 6). This argues that high scores on Conscientiousness and Agreeableness could be associated with both lower and higher MARS scores.

**Table 5:** Comparisons between high scorers on Conscientiousness with reported high or low MARS¤ scores.

Variable	High C# and Low MARS¤	High C# and High MARS¤	P
	(n = 18) Mean (SD)*	(n = 236) Mean (SD)*	
**Age**	47.11 (9.05)	53.95 (10.62)	0.008
**Neuroticism**	17.06 (9.54)	12.59 (7.31)	0.015

¤ = Medication Adherence Report Scale,

# = Conscientiousness,

* = Standard deviation.

**Table 6:** Comparisons between high scorers on Agreeableness with reported high or low MARS¤ scores.

Variable	High A# Low MARS¤	High A# High MARS¤	P
	(n = 10) Mean (SD)*	(n = 99) Mean (SD)*	
**Openness to experiences**	32.80 (7.46)	27.31 (6.10)	0.009
**Conscientiousness**	32.40 (6.70)	37.16 (5.62)	0.014

¤ = Medication Adherence Report Scale,

# = Agreeableness,

* = Standard deviation.

The multiple regression with a spline function relating Openness to experience vs. MARS, revealed that Openness scores below 23 increased MARS scores slightly, while scores above 23 resulted in a slight reduction in MARS scores (Figure 2d). The relation between Extraversion and MARS was almost horizontal, arguing that Extraversion could not be regarded as an influential factor of adherence behaviour in the present study (Figure 2e).

### Non-response study

Results from the non-response study indicated that study participants reported chronic disease to a greater extent than non-responders (P = 0.032); no differences relating to age and education level were found. As hypothesised, some differences in personality scores were identified; non-responders scored higher for Neuroticism (P = 0.009), Extraversion (P<0.001), and Agreeableness (P<0.001).

## Discussion

The results of the current study argue that multiple relationships between personality traits and adherence to medication are present in individuals with chronic disease. Three of the five personality traits, Neuroticism, Agreeableness and Conscientiousness, influenced reported medication adherence behaviour. Published studies to date have presented associations with one [11]–[13], [16], [18] or two personality traits [14]. However, these studies have been based on clinical samples, and not, as in this study, on a random population sample, which avoids selection bias [25]. In particular, we have demonstrated that Neuroticism was negatively related to medication adherence, while Agreeableness and Conscientiousness were positively related to medication adherence. However, non-linear analyses showed that some individuals who rate high on Conscientiousness and Agreeableness deviate from the general adherence behaviour by reporting lower adherence scores. Such deviation was closely associated with other personality traits.

A high score on Neuroticism is related to *“a worrying kind of person”* [7]. Previous studies have recognised that individuals greatly influenced by this personality trait tend to have behaviour that is riskier to their health, such as tending to smoke [26] and adhering inappropriately to disease management suggestions [14]. Despite this health risk behaviour, this personality trait is associated with frequent health care utilisation, and such individuals are inclined to be more attentive to somatic symptoms [27]. Our findings confirming low reported adherence to medication in chronically ill individuals with this personality trait, further emphasise the importance of identifying patients with such personality characteristics. A linear analysis did not show a close relationship between the score for Neuroticism and MARS, arguing that factors, other than Neuroticism alone, influence adherence behaviour, which is indeed confirmed in additional analyses of other personality traits.

Conscientiousness is the personality trait that is most frequently reported to be associated with high adherence to prescribed medication [11]–[13].This is confirmed by the high reported rate of adherence in the current random population sample of individuals with chronic disease. However, non-linear analyses indicated that the highest scores on Conscientiousness were associated with a marked reduction in adherence scores. This shift in the relationship to high adherence in individuals who scored very high on this personality trait may be explained by their tendency to put high trust in their own ability to manage their lives [7]. Thus, it is possible that these high scorers trust their own ability to make judgements about their medication needs, and could thus adjust medication instead of adhering to a prescription. Another explanation could be that these individuals scored higher on Neuroticism or that they were younger than those who reported higher adherence scores. Indeed, in the present study, lower age and higher scores on Neuroticism were identified separately as factors influencing low medication adherence in these high scorers. Thus, no single personality trait could explain a tendency for low or high adherence to prescribed medication.

The non-linear relationship between Agreeableness and adherence scores showed that this personality trait had a positive impact on adherence only up to a certain level, after which adherence scores declined for those with high score of Agreeableness. A possible explanation could be that some individuals scoring high on Agreeableness have an altruistic disposition [7] and tend to prioritise the needs of others before their own. An alternative explanation for the observed lower adherence scores may be that these individuals were rated lower on Conscientiousness, which we indeed could confirm [11]–[13]. Again, these data argue that a complex relationship exists between personality traits and adherence to medication, with multiple traits influencing patient behaviour.

The present study did not show any association between Extraversion and Openness to experiences and adherence behaviour. A previous study presented associations between Extraversion and exercise adherence [17], but only one patient-based study has identified a relationship between Extraversion and adherence to treatment with antidepressants [18]. An explanation for the discrepancy between the previous and present studies might be that individuals who score high on Extraversion are prone to a physically active lifestyle [28]. Furthermore, it cannot be excluded that personality traits may have a slightly different influence on specific health behaviours in patients with different chronic diseases.

The strength of the present study is that it is based on random population sample, which increases its overall validity. There are of course several limitations, including the relatively low response rate and the MARS questionnaire which shows quite a low variance. The reported mean adherence scores were skewed towards higher scores, indicating that the MARS did not fully discriminate all levels of treatment adherence. A positive skew has been documented in other studies that have used the MARS instrument [29]–[30]. Thus, to fully explore the relationship between personality traits and other factors that may influence adherence to medication, advanced prospective and objective measures of adherence have been proposed, although measuring adherence is complicated [6]. A variant of MARS for asthma has indeed been found to predict adherence using objective electronic measurement of adherence [31], whereas another method of self-reporting failed to predict more objective adherence [32].

The use of spline functions, which fit the data more closely than linear regression analyses, identified non-linear associations [23]. Without regression modelling with a spline function, some of our findings could have been overlooked, in particular, those that demonstrate that multiple personality traits influence adherence behaviour independently, especially in individuals who score very highly on Conscientiousness and Agreeableness.

In epidemiological studies using mailed questionnaires, it is important to determine whether differences exist between participants and non-responders. In the current analysis, we found that individuals with chronic disease seemed to have a greater tendency to participate, but there was no difference relating to age or level of education, arguing that the data we present are indeed representative of individuals with chronic illness. Not surprisingly, some differences in personality traits were discovered between participants and non-responders. In fact, non-responders had higher scores on Neuroticism, Agreeableness and Extraversion. The loss of some individuals with high scores on Neuroticism and Agreeableness did not prevent the current study from detecting associations between these traits and adherence to medication.

This study shows that personality traits seem to be related to reported adherence to treatment. Although additional factors, such as beliefs about medication, patient self-efficiency, and illness perception, should be considered in a clinical setting, it can be hypothesised that low adherence behaviour could be predicted by personality trait analysis. In view of the increasing level of chronic disease in the ageing European population and the economic significance of the problem of non-adherence to treatment, analysis of personality traits could be used to guide efforts to educate and support patients with a high risk of poor adherence by identifying these individuals.
